# Evaluation of the Effect of Probiotic *Bacillus coagulans* Unique IS2 on Mutans Streptococci and Lactobacilli Levels in Saliva and Plaque: A Double-Blind, Randomized, Placebo-Controlled Study in Children

**DOI:** 10.1155/2020/8891708

**Published:** 2020-12-29

**Authors:** M. Ratna Sudha, Jayanthi Neelamraju, M. Surendra Reddy, Manoj Kumar

**Affiliations:** ^1^Centre for Research and Development, Unique Biotech Ltd., Plot No. 2, Phase-II, Alexandria Knowledge Park, Hyderabad, Telangana 500 078, India; ^2^Department of Pedodontics and Preventive Dentistry, SVS Institute of Dental Sciences, Mahbubnagar, Telangana, India

## Abstract

**Background:**

Probiotic organisms are known to reduce caries causing microorganisms *mutans streptococci* and *lactobacilli*. *Aim of the Study*. To evaluate the effect of probiotic *Bacillus coagulans* Unique IS2 on mutans streptococci and lactobacilli levels in saliva and plaque in children.

**Introduction:**

Dental caries or tooth decay is because of the demineralization of the tooth enamel leading to the breakdown of the enamel causing cavities to be formed. Demineralization of the tooth happens because of the acid secreted by bacteria like *mutans streptococci* and *lactobacilli*. It is now suggested that probiotic usage prevents the overgrowth of these pathogenic microbes, thereby reducing caries activity. *Methodology*. In this double-blind, randomized, placebo-controlled study, 48 children with ages ranging from 5 to 15 years were divided into two groups, the probiotic and placebo groups. Chewable tablets with and without probiotic *Bacillus coagulans* Unique IS2 were administered for two weeks. Stimulated saliva samples and plaque were collected at baseline and at the end of 14 days to measure the pH, *mutans streptococci,* and *lactobacilli* count of saliva and plaque using chairside kits.

**Results:**

A statistically significant reduction in *mutans streptococci* and *lactobacilli* counts of both saliva and plaque samples was observed in the *B*. *coagulans* Unique IS2 treated group after 14 days of administration compared to the baseline and placebo group (using paired *t*-test).

**Conclusion:**

Probiotic *Bacillus coagulans* Unique IS2 (2 billion cfu) chewable tablet is effective in reduction and inhibition of caries causing *mutans streptococci* and *lactobacilli* levels in saliva and plaque in children.

## 1. Introduction

Oral diseases are a major health burden globally and people of all ages throughout their lifetime are affected [1]. Some of the common diseases that affect oral health are tooth decay (cavities), gingivitis, gum (periodontal) disease, halitosis (bad breath), and oral cancer.

It is estimated that oral diseases affect close to 3.5 billion people worldwide, with dental caries (tooth decay) being the most common condition [2]. It is estimated that 2.3 billion people globally suffer from caries of permanent teeth and more than 530 million children suffer from caries of primary teeth [3]. The percentage of school children affected by dental caries is 60%–90% and this is common for both developed and developing countries [4]. Some of the causes for dental caries include consumption of foods and beverages high in sugars, lack of oral hygiene, and absence of yearly dental check-ups. In developing countries where there is a lack of basic health facilities, oral health is grossly neglected leading to the high prevalence of dental caries especially in children [5]. Dental caries is a cause of pain, discomfort with enhanced sensitivity to cold and hot beverages apart from the loss of aesthetical appearance. The cost of dental treatments is high and hence prevention of dental caries by saving the teeth from decay is highly recommended instead of treatments aimed at drilling out the decay and putting in a filling (restoration) made from various materials [6]. Various methods to prevent dental caries have been employed [7] and these include treatment with fluoride (water fluoridation and fluoride toothpaste) and removal of dental plaque by use of antibacterial mouth rinses. Other methods to prevent dental caries include the application of pit and fissure sealants on susceptible pits and fissures of the tooth, but these forms of treatment are again costly. The disadvantage of fluoride treatment is that ingestion of excess fluoride can cause skeletal and dental fluorosis. The disadvantage of antibacterial mouth rinses is that even the good bacteria in the oral cavity are killed creating open, noncompetitive surfaces for pathogens to repopulate the oral cavity; moreover, long-term use can cause staining of teeth. Previous studies have suggested that probiotics can be effective in selectively inhibiting oral pathogens and modulating the microbial composition of dental plaque thereby reduce the incidence of dental caries [7–9]. The advantage of probiotics as compared to other preventive methods is that they are generally regarded as safe and have additional health benefits like maintaining a healthy gut. The main pathogen involved in the causation of dental caries is *Streptococcus mutans* [10]. Probiotics are live microorganisms that when ingested in adequate quantities confer a health benefit to the host. Probiotics in general have been associated with immunity and gut health, and attention has mostly focused on the prevention or treatment of gastrointestinal infections [11]. Probiotics are now known to be efficacious in the treatment of various other diseases which include cardiovascular health, diabetes, weight management, cancer, brain health (anxiety and depression, stress), urogenital health, skin diseases, allergies, and oral health [12–16].

The role of probiotics in oral health management (caries, gingivitis, and periodontal management) is increasingly being recognised with the mechanisms of action considered to be antagonistic activity against pathogens, coaggregation and growth inhibition of pathogens, bacteriocin, organic acid, and hydrogen peroxide production, and interaction with oral epithelium resulting in the reduction of the pathogenicity and cariogenic potential of biofilm microorganisms [17, 18]. Immunity also appears to have a role with both innate and adaptive immune responses involved in combatting oral diseases [19].

Several studies indicate that the consumption of probiotics can inhibit the levels of *mutans streptococci* in saliva [20–23]. Strains of *Lactobacillus casei*, *L*. *rhamnosus*, *L*. *reuteri*, and *Bifidobacterium* spp. have demonstrated the ability to inhibit cariogenic bacteria and their colonisation and thus prevent dental caries [24]. A previous study with the spore-forming probiotic strain *Bacillus coagulans* found that it was effective in reducing the *mutans streptococci* count [25]. Lactic acid bacteria, the *lactobacilli* spp. in plaque produce lactic acid and other organic acids from the fermentation of sugars (from the diet of the host) which lead to the progression of dental caries. It is also suggested that in caries-free children, *lactobacilli* have been found to be largely absent [26]. It was thus of interest to measure the pH, *lactobacilli*, and *mutans streptococci* levels in both saliva and plaque of cariogenic children. Since the effects of probiotics are strain-specific, the objective of the present study was to assess the anticariogenic potential of *Bacillus coagulans* Unique IS2 in dental caries. *B*. *coagulans* Unique IS2 is a safe, well-characterized, and stable strain [27, 28], with documented efficacy in restoring digestive health [29–33], reduction of bacterial vaginosis [34], and reduction of side effects of liver cirrhosis [35].

The advantage of *B*. *coagulans* Unique IS2 is that it is a spore-forming probiotic strain and hence very resilient to harsh manufacturing processes which allows it to be incorporated in any dosage form without loss of viability unlike the vegetative *lactobacillus and Bifidobacterium* spp. which cannot withstand harsh processes and also require refrigeration. As probiotic effects are not only strain-specific but also dependent on dosage and dosage forms, the aim of this study was to evaluate the efficacy of *B*. *coagulans* Unique IS2 chewable tablets on cariogenic organisms in children prone to dental caries.

## 2. Methods

### 2.1. Study Design

This double-blind, randomized, placebo-controlled study was conducted at the Department of Pedodontics and Preventive Dentistry, SVS Institute of Dental Sciences, Mahabubnagar, Telangana, India.

The study was conducted in accordance with the National Ethical Guidelines for biomedical and health research involving human participants (Indian Council of Medical Research) and the principles of the Declaration of Helsinki. The Ethical Committee of the SVS Institute of Dental Sciences approved the study before initiation of the trial. The trial was also registered with the Clinical Trial Registry- India (CTRI Reg No. CTRI/2017/07/009072).

### 2.2. Study Population

**Figure 1:**
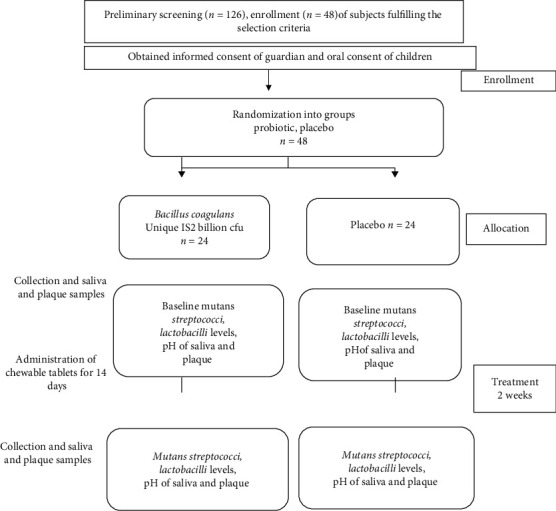
Flow chart of study design.

126 children were screened, and based on the inclusion and exclusion criteria, 48 children were selected. The sample size of 24 children per group was based on the confidence level (95%) and margin of error (1%). There were no dropouts during the study; therefore, all 48 children completed the study (Figure 1). The mean age of the patients was 8.5 ± 1.6 years (range of 5–15 years). 20 (41.67%) patients were males and 28 (58.33%) patients were females. The children were divided by randomization (computer-generated random numbers) into two equal groups of 24 each—the probiotic or placebo-treated group. The probiotic group consumed *B*. *coagulans* Unique IS2 chewable tablets (2 billion cfu) once daily for a period of fourteen days whereas the placebo group was administered placebo chewable tablets for the same length of time. Both the placebo and *B*. *coagulans* Unique IS2 chewable tablets were manufactured by Unique Biotech Ltd., Hyderabad, India. The placebo chewable tablets differed from the probiotic tablets in that they lacked the active ingredient, *B*. *coagulans* Unique IS2; otherwise, they were similar with respect to shape, size, and colour to the probiotic chewable tablets. All subjects in the study were of Indian origin and consumed a similar Indian diet of rice, dhal, vegetables, and chapatis (Indian bread). Both the groups were similar with respect to age and sex of the subjects.

Prior to the initiation of the study, children who were selected after screening (on screening day) were provided instructions on oral hygiene and asked to maintain oral health care during the period of treatment. Instructions were provided on the right method of brushing and the need to brush twice daily and rinse vigorously after meals. They were however instructed not to brush their teeth 24 hours prior to Visit 1 (next day after screening and selection) and Visit 2 (after 14 days of treatment) in order to enable plaque collection with ease.

### 2.3. Selection Criteria

#### 2.3.1. Inclusion Criteria

The study included children of either sex between 5 and 15 years of age with increased caries risk (DMFS/defs ≥ 5). Other inclusion criteria: children who had not consumed antibiotics or probiotics in any form three months prior to the study, children who were willing to chew the tablets and participate in the study, and parents of the subjects were willing to give written informed consent and follow study procedures.

#### 2.3.2. Exclusion Criteria

Exclusion criteria included children with severe infections, diseases affecting the whole body, weakened immune systems, and congenital abnormalities; children on fluoride therapy; children using xylitol gum, probiotic products, antibiotics, or corticosteroids within three months of the study initiation; and those not willing to participate in the study.

#### 2.3.3. Randomization

Enrollment of the children who complied with the inclusion/exclusion criteria was done after obtaining signed, written informed consent from the parents. Block randomization was used to divide the children into two treatment arms, the probiotic and placebo-treated groups. Randomization numbers were generated using SAS programming (Statistical Package for Social Sciences, version 18.0, SPSS Inc., Chicago, Illinois, USA). The sealed opaque envelope method was used to keep the study double-blinded. The envelopes with the assignment of probiotic or placebo treatment were provided to the clinical site. The investigators in turn disbursed the chewable tablets (probiotic or placebo) to patients based on the randomization numbers.

### 2.4. Study Follow-Up Visits and Treatments

*B*. *coagulans* Unique IS2 or placebo chewable tablet was administered once daily for fourteen days. As this was an outpatient study, two visits (Visit 1 and Visit 2) were recorded. Visit 1 was the next day after screening and selection. After collection of saliva and plaque samples for baseline values, treatment was initiated on the same day (Day 1). Visit 2 was the follow-up after the end of treatment (the next day after 14 days of treatment). For both visits, children were instructed not to brush their teeth 24 hrs prior to the visit in order to enable plaque collection with ease.

### 2.5. Efficacy Parameters

The decrease in the levels of *mutans streptococci* in saliva and plaque was the primary efficacy parameter studied.

Secondary efficacy parameters assessed were measurement of *lactobacilli* levels and pH in both saliva and plaque.

### 2.6. Collection of Saliva Samples

Stimulated saliva samples were collected in a graded tube to measure pH, *mutans streptococci*, and *lactobacilli* levels according to manufacturer's instructions (CRT, Ivoclar Vivadent AG, Schaan, Liechtenstein). Salivary samples were collected at both visits between 9 a.m. and 10 a.m. to avoid any diurnal variations. Children were asked to rinse their mouth thoroughly with distilled water for one minute and then chew on a piece of paraffin wax provided by the manufacturer for five minutes (CRT, Ivoclar Vivadent AG, Schaan, Liechtenstein). The saliva samples were then collected for analysis.

### 2.7. Collection of Plaque Samples

Sterile toothpicks were used to collect supragingival plaque from all the teeth. Samples were then transferred to a sterile test tube and diluted in a 1 : 10 ratio for all the estimations.

### 2.8. Estimation of Salivary and Plaque *Mutans Streptococci* and *Lactobacilli*

The counts of salivary and plaque *mutans streptococci* and *lactobacilli* were estimated with the chairside test (CRT Bacteria, Ivoclar Vivadent AG, Schaan, Liechtenstein) (Table 1) according to the manufacturer's instructions [36].

After identification and counting of the colonies, conversion to scores was done according to the manufacturer's instructions. Scores ranged from 1 to 4 for both *mutans streptococci* and *lactobacilli* as per the number of colony forming units (cfu/ml) (Table 1).

### 2.9. Evaluation of Salivary and Plaque pH

The pH of saliva and plaque was measured using the chair-side test strip, details of which are provided in an earlier study [37].

### 2.10. Statistical Analysis

Paired *t*-test was used to compare baseline values to the end of treatment values. The two groups were compared for changes from baseline values using one sample *t*-test and 95% CI. Measurement data was expressed as means with SD. Values with *P* < 0.05 were considered significant.

## 3. Results

### 3.1. Primary Efficacy Parameters

#### 3.1.1. Salivary and Plaque *Mutans Streptococci* Levels

**Figure 2:**
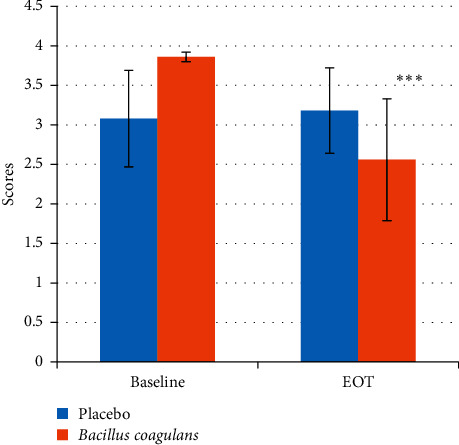
Salivary *mutans streptococci* scores at baseline and EOT. Values are mean ± s.d. ^*∗∗∗*^*p* value <0.001.

**Figure 3:**
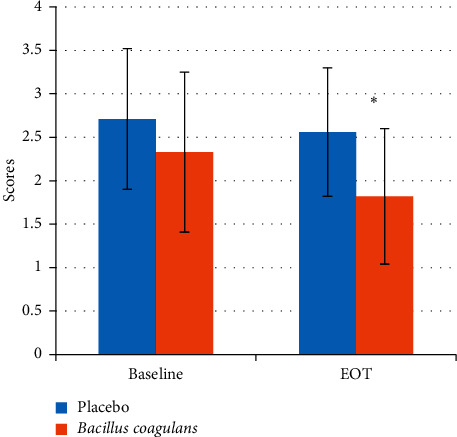
Plaque *mutans streptococci* scores at baseline and EOT. Values are mean ± s.d. ^*∗*^*p* value <0.05.

There was a significant reduction (*P* value < 0.001) in the salivary *mutans streptococci* levels (as indicated by the scores) in the probiotic treated group after 14 days as compared to the baseline (3.86 ± 0.06 to 2.56 ± 0.77) whereas there was no significant change in the placebo group (3.08 ± 0.61 to 3.18 ± 0.54) (Figure 2, Table 2). Similarly, in the plaque samples, there was a significant reduction (*P* value < 0.05) in the *mutans streptococci* levels in the probiotic treated group after 14 days as compared to the baseline (2.33 ± 0.92 to 1.82 ± 0.78) whereas there was no significant change in the placebo group (2.71 ± 0.81 to 2.56 ± 0.74) (Figure 3, Table 3).

#### 3.1.2. Salivary and Plaque *Lactobacilli* Scores

**Figure 4:**
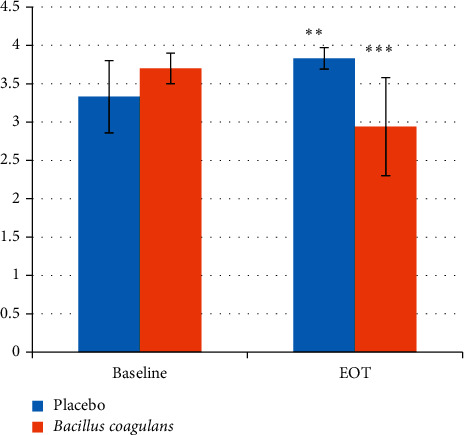
Salivary *lactobacilli* scores at baseline and EOT. Values are mean ± s.d. ^*∗∗*^*p* value <0.01. ^*∗∗∗*^*p* value <0.001.

**Figure 5:**
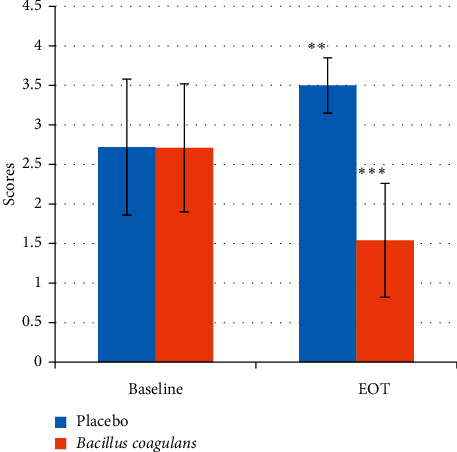
Plaque *lactobacillus* scores at baseline and EOT. Values are mean ± s.d. ^*∗∗*^*p* value <0.01. ^*∗∗∗*^*p* value <0.001.

There was a significant reduction (*P* value < 0.001) in the salivary *lactobacilli* levels (as indicated by the scores) in the probiotic treated group after 14 days as compared to the baseline (3.70 ± 0.2 to 2.94 ± 0.64). On the contrary, in the placebo-treated group, a significant increase in the *lactobacilli* levels (3.33 ± 0.47 to 3.83 ± 0.14) was observed (Figure 4, Table 4). In the plaque samples, the same trend was observed with a significant decrease in *lactobacilli* levels in the probiotic treated group (2.71 ± 0.81 to 1.54 ± 0.72) and a significant increase in the *lactobacilli* levels in the placebo group (2.71 ± 0.86 to 3.50 ± 0.35) (Figure 5, Table 5).

#### 3.1.3. pH of Saliva and Plaque Samples

There was no change in the pH of the saliva samples either with probiotic or placebo treatment with pH remaining in the range of 7.6–7.8 (*P*=0.553). Similarly, there was no change in the pH of the plaque samples with either treatment with pH in the range of 6.0–6.3 (*P*=0.5030; data not shown).

## 4. Discussion

The ability of chewable tablets containing the probiotic *B*. *coagulans* Unique IS2 in lowering the levels of microbes involved in the causation of dental caries, *mutans streptococci*, and *lactobacilli* was evaluated in this double-blind, placebo-controlled study. The subjects included in the study had a propensity towards dental caries (DMFS/defs ≥ 5). It is suggested that the prevalence of *mutans streptococci* and *lactobacilli* is increased in plaque and saliva in children with increased caries activity and probiotics can play a role in inhibiting the cariogenic bacteria. Probiotics can therefore be considered as an important alternative therapy for the replacement of pathogenic microorganisms [38–40].

Chewable tablet was chosen as the dosage form for the probiotic supplement as it allows for the probiotic strain to remain longer in the mouth and populates the oral cavity while chewing. The results indicated that daily consumption of *B*. *coagulans* Unique IS2 probiotic chewable tablets for two weeks effectively reduced the *mutans streptococci* and *lactobacilli* counts in both saliva and plaque samples. The duration of two weeks of treatment was chosen based on earlier studies where the efficacy of probiotic strains was demonstrated with the same timeline [25, 41, 42]. This decrease in the levels of *mutans streptococci* is similar to the findings of some studies [25, 43, 44]. To the best of our knowledge, the efficacy of *B*. *coagulans* on the levels of salivary or plaque *lactobacilli* has not been measured. Our study has reported that there is a significant decrease in both the salivary and plaque *lactobacilli* with *B*. *coagulans* Unique IS2 treatment; however, there was no change in the pH of saliva and plaque samples with either probiotic or placebo treatment. With the significant decrease of *lactobacilli* spp. in the saliva and plaque samples of the *B*. *coagulans* Unique IS2 treated group, one would expect an increase in the pH, but this was not observed and could be attributed to the buffering capacity of saliva. A similar observation was made by Villavicencio et al. [40] wherein there was a significant decrease in the *lactobacilli* spp. in the saliva of children on probiotic treatment but no significant change of pH.

The present report evaluated a selected population of patients, that is, children prone to dental caries. The microbiology of the oral environment can however be altered in other populations by other variables such as restorative frameworks [45] and orthodontic appliances [46]. Additionally, wear can alter surface characteristics of enamel [47] and composite materials [48]and this could have an important influence on bacterial colonisation. Therefore, further studies are needed in order to gain an insight into the role of *Bacillus coagulans* Unique IS2 in altering the oral microflora in varying oral health conditions. It is known that the genetic make-up of saliva may have an influence on the *mutans streptococci* levels. Differences in susceptibility to dental caries occur even under the similar, controlled conditions because of genetic variations. Some environmental factors are potentially more cariogenic for some individuals than for others [49]. Most genetic studies have focused on detecting a genetic factor contributing to caries by testing genetic variation, such as single-nucleotide polymorphisms (SNPs) in specific genes, for an association between variants at a genetic locus and caries. These genes are grouped into categories based on the factor influencing dental caries. The major candidate gene categories include enamel formation genes, immune response genes, genes related to saliva, and genes related to taste and dietary habits [50].

*B*. *coagulans* Unique IS2 has previously been found to be efficacious in the treatment of diarrhoea [29], constipation [30], and irritable bowel syndrome (IBS) in both adults and children [31, 32]. In the study with IBS in children, the dosage from *B*. *coagulans* Unique IS2 (2 billion cfu) was in the form of chewable tablets, the same as used for the present study.

In conclusion, a fourteen-day administration of probiotic *B*. *coagulans* Unique IS2 in the form of chewable tablets is able to reduce cariogenic bacteria. A reduction of salivary and plaque mutans *streptococci* and *lactobacilli* concentration in children is an indication of reduced caries risk. The limitation of the present study is that it was done with a small population size. Studies with a larger sample size and a longer follow-up are however needed to make any statistically conclusive assertions.

## Figures and Tables

**Table 1:** 

Scoring for *mutans streptococci*				
Score	1	2	3	4
Cfu/ml	<10^3^	10^3^<10^5^	10^5^–10^6^	>10^6^

Scoring for *lactobacilli*				
Cfu/ml	≤10^3^	10^4^	10^5^	≥10^6^

**Table 2:** Salivary *mutans streptococci* scores at baseline and EOT.

		Baseline	End of treatment
Placebo	Min value	2.39	2.39
	Max value	3.78	3.89
	Median	3.54	3.70
	Mean	3.08	3.18
	s.d.	0.61	0.54

*Bacillus coagulans* Unique IS2	Min value	3.75	1.13
	Max value	3.96	3.89
	Median	3.85	2.58
	Mean	3.86	2.56
	s.d.	0.06	0.77

**Table 3:** Plaque *mutans streptococci* scores at baseline and EOT.

		Baseline	End of treatment
Placebo	Min value	1.84	1.72
	Max value	3.58	3.4
	Median	1.87	1.72
	Mean	2.71	2.56
	s.d.	0.81	0.74

*Bacillus coagulans* Unique IS2	Min value	1.35	1.03
	Max value	3.28	2.62
	Median	1.36	2.51
	Mean	2.33	1.81
	s.d.	0.92	0.78

**Table 4:** Salivary *lactobacilli* scores at baseline and EOT.

		Baseline	End of treatment
Placebo	Min value	2.61	3.35
	Max value	3.9	3.92
	Median	3.61	3.88
	Mean	3.3	3.83
	s.d.	0.47	0.14

*Bacillus coagulans* Unique IS2	Min value	3.28	2.24
	Max value	3.93	3.62
	Median	3.80	3.51
	Mean	3.70	2.94
	s.d.	0.2	0.64

**Table 5:** Plaque *lactobacillus* scores at baseline and EOT.

		Baseline	End of treatment
Placebo	Min value	1.84	2.72
	Max value	3.57	3.82
	Median	3.55	3.65
	Mean	2.72	3.50
	s.d.	0.86	0.35

*Bacillus coagulans* Unique IS2	Min value	1.89	0.81
	Max value	3.52	2.26
	Median	3.5	2.25
	Mean	2.71	1.54
	s.d.	0.81	0.72

## Data Availability

The data used to support this study were obtained from the Department of Pedodontics and Preventive Dentistry, SVS Institute of Dental Sciences, Mahbubnagar, Telangana, India.
